# Collegiate Education and the Medical Profession

**Published:** 1890-08

**Authors:** 


COLLEGIATE EDUCATION AND THE MEDICAL PROFESSION.—The question of a college education in its relation to business occupations and to the liberal professions is again exciting deep interest. The discussions, at least, show a wide-spread feeling that there is something wrong in the present methods and tendencies of some of our colleges.

The doctor has a special interest in this subject, for these methods, as pursued in some quarters, are tending more and more to take away the possibility of any large proportion of young men going through college before they study medicine.

In a recent address delivered by Dr. D. B. St. J. Roosa, before the Yale Association of Fairfied county, this matter was most forcibly presented, and the difficulties which have been raised by modern collegiate curriculm logically set forth. There is no combating Dr. Roosa's conclusious, which are substantially in harmony with what we have before presented to readers of the *Medical Record.*

It is desirable for those who are to follow a liberal profession to have a liberal education. It not only broadens the mind, trains the faculties, but gives one larger capacities for enjoying life. Admitting this, however, it is equally true that few prospective doctors can afford to go to a college from which they can only graduate at the age of twenty-two or twenty-three. For they cannot then complete their medical education and get to practice before they are twenty-seven or eight; and they are not earning a living until they are thirty.

It seems to be agreed that a sufficiently liberal education ought to be acquired by the age of twenty, or at the end of the sophomore year in a university like Harvard. At this time special lines of work should be begun, bearing reference to their future calling in life. In other words, as Dr. Roosa puts it, college education should end at twenty, and university education begin at that time. The system which has gradually been evolved at Harvard is utterly unsuited to the great majority of American students. "It is neither fish, flesh, fowl, nor good red-herring," says our lecturer most truthfully; it is too much for a college education and not enough for a university education.

What shall be done then? We can let our large so-called universities settle it among themselves. There are enough rich people in the country to no doubt support such institutions, if they do not wish to change. But, meanwhile, young men who want to study medicine, or law, or engineering, or go into business, and also go through college, should attend those institutions which are in fact colleges, and which get them through by the time they are twenty years of age.

As a matter of fact this is being done. There are very few Harvard graduates in the Harvard Medical School, whereas in Dartmouth Medical College, for example, nearly one-third of the students are graduates of the Academic Department. We advise young men to get a good, sound, liberal education at a small and a genuine classical college. Many can finish this even before they are twenty. Then they are none too old to start in with their professional studies.—*Editorial in Medical Record, June, 1890.*

